# Complete Genome Sequence of Mycobacterium shigaense

**DOI:** 10.1128/genomeA.00552-18

**Published:** 2018-06-21

**Authors:** Mitsunori Yoshida, Hanako Fukano, Yoshitoshi Ogura, Yuko Kazumi, Satoshi Mitarai, Tetsuya Hayashi, Yoshihiko Hoshino

**Affiliations:** a Department of Mycobacteriology, Leprosy Research Center, National Institute of Infectious Diseases, Tokyo, Japan; b Department of Bacteriology, Faculty of Medicine Sciences, Kyushu University, Fukuoka, Japan; c The Research Institute of Tuberculosis, Japan Anti-Tuberculosis Association, Tokyo, Japan

## Abstract

Mycobacterium shigaense is a slowly growing scotochromogenic species and a member of the Mycobacterium simiae complex group. Here, we report the complete sequence of its genome, comprising a 5.2-Mb chromosome. The sequence will represent the essential data for future phylogenetic and comparative genome studies of the Mycobacterium simiae complex group.

## GENOME ANNOUNCEMENT

Mycobacterium shigaense was reported by Nakanaga et al. in 2012 as a causative agent of skin lesions in an immune-suppressed patient (1). After that, a total of six patients were diagnosed and another three cases have already been reported, and we recently summarized the characteristics of M. shigaense (2–5). Notably, among four out of six patients (66.7%), the place they live is the Shiga prefecture, for which this strain is named. The first clinical isolate (JCM 32072^T^) is a reference strain of “M. shigaense.” Here, we report the complete genome sequence of JCM 32072^T^.

The strain was grown with Middlebrook 7H9 medium. DNA was purified with the standard phenol-chloroform method. The genome sequence was determined using PacBio reads (149,374 subreads) obtained with an RS II system (Menlo Park, CA, USA), and Illumina 150 × 2 paired-end reads (692,827 reads) obtained with a MiSeq sequencer (Illumina, San Diego, USA) were used for correction of sequence and assembly errors (6–10). The reads were assembled into one contig by using the HGAP/Quiver assembly approach (11) and circularized using Circlator (12). Alignments produced by mapping Illumina reads to the assembly using the Burrows–Wheeler aligner (13) were used for correction of sequence and assembly errors with Pilon (14). Automated annotation was carried out with the DDBJ Fast Annotation and Submission Tool (DFAST) (https://dfast.nig.ac.jp) (8, 9, 15, 16). Average nucleotide identity (ANI) was calculated by JSpeciesWS (17).

The chromosome of JCM 32072^T^ is 5,232,660 bp in length with 67.3% G+C content. The average nucleotide identities for this strain were between 76 and 81% of those for the M. simiae complex group (3). The chromosome contains 4,920 predicted protein-coding sequences (CDS), which is much fewer than those of the M. simiae strain MO323 (5,664 CDS). The genome sequence of M. shigaense JCM 32072^T^ will represent the essential data for future phylogenetic and comparative genome studies and will be useful for better understanding the evolution of the M. simiae complex group.

### Accession number(s).

This complete genome sequence has been deposited at DDBJ/ENA/GenBank under the accession no. AP018164.
